# Magnetoelectric Response in Multiferroic SrFe_12_O_19_ Ceramics

**DOI:** 10.1371/journal.pone.0167084

**Published:** 2016-12-09

**Authors:** Guolong Tan, Yao Huang, Haohao Sheng

**Affiliations:** State Key Laboratory of Advanced Technology for Materials Synthesis and Processing, Wuhan University of Technology, Wuhan, China; Institute of Materials Science, GERMANY

## Abstract

We report here realization of ferroelectricity, ferromagnetism and magnetocapacitance effect in singleSrFe_12_O_19_ceramic at room temperature. The ceramics demonstrate a saturated polarization hysteresis loop, two nonlinear I-V peaks and large anomaly of dielectric constant near Curie temperature, which confirm the intrinsic ferroelectricity of SrFe_12_O_19_ ceramicswith subsequent heat-treatment in O_2_atmosphere. The remnant polarization of the SrFe_12_O_19_ ceramic is estimated to be 103μC/cm^2^. The ceramic also exhibits strong ferromagnetic characterization, the coercive field and remnant magnetic moment are 6192Oe and 35.8emu/g, respectively. Subsequent annealing SrFe_12_O_19_ ceramics in O_2_ plays a key role on revealing its intrinsic ferroelectricity and improving the ferromagnetism through transforming Fe^2+^ into Fe^3+^. By applying a magnetic field, the capacitance demonstrates remarkable change along with B field, the maximum rate of change in *ε (*Δε(B)/ε(0)) is 1174%, which reflects a giant magnetocapacitance effect in SrFe_12_O_19_. XPS and molecular magnetic moment measurements confirmed the transformation of Fe^2+^ into Fe^3+^ and removal of oxygen vacancies upon O_2_ heat treatment. These combined functional responses in SrFe_12_O_19_ ceramics opens substantial possibilities for applications in novel electric devices.

## Introduction

Multiferroics is a class of functional materials that simultaneously exhibit ferroelectricity and ferromagnetism in a single structure [1–3]. They can demonstrate not only the magnetic or electric polarization but also the desired magnetoelectric (ME) coupling between the two orders leading to multifunctional performance, such as electric field controlled magnetic data storage or vice versa [4]. This unique coupling feature has a tremendous impact on technology, with potential application for spintronic devices, solid-state transformers, high sensitivity magnetic field sensors, and actuators [5]. As part of the technological drive toward device miniaturization, considerable effort has been devoted to the combination of electronic and magnetic properties into one multifunctional material, i.e., a single device component that can perform more than one task [5–9]. Such idea of combining two orders in one single compound has stimulated a vast research of new multiferroic materials [10–16]. Except the most heavily reported multiferroic compounds, such as BiFeO_3_ [1, 4, 5], TbMnO_3_ [17] and DyMnO_3_ [18], some ferrites with hexagonal structures, termed as hexaferrites, have been found to show such ME effects as magnetic field induced ferroelectrics and drawn our attention [19–21]. Although several Y-type and Z-type hexaferrites were reported to demonstrate some ME effects and remarkable changes in polarization upon a magnetic field [22–25], the ME effect in these ferrites is small, their pure electric polarization or ferroelectric features (P-E loops) arestill absent and the magnetism is weak [26]. For applications, however, it will be necessary to generate simultaneously ferroelectricity and ferromagnetism, together with giant ME effects in one single compound at room temperature. Hence, it is a long standing challenge in the research of multiferroics to improve the operating temperature [26] and the ME sensitivity [21, 27].

M-type lead hexaferrite (PbFe_12_O_19_) has demonstrated coexistence of large ferroelectricity and strong ferromagnetism at room temperature [28. 29]. However, lead (Pb) is a kind of toxic element and PbFe_12_O_19_ is not an environment-friendly material. SrFe_12_O_19_, instead, is a lead-free M-type hexaferrite and environment-friendly. It has attracted a lot of attention because of its non-toxicity, excellent magnetic properties and wide application in various fieldsuch as magnetic recording and high-frequency devices [30, 31]. Recently, the dielectric and ferroelectric features of M-type hexaferrites, such as BaFe_12_O_19_ single crystal, have attracted some attentions [32–34]. However, the authors claimed that M-type barium hexaferrite, belongs to quantum paraelectrics due to the electric dipole of a FeO_5_ bipyramid [32,33]. This kind of conclusion conflicts with the reported intrinsic ferroelectricity of PbFe_12_O_19_ [29]. After careful analysis of the structure data of the BaFe_12_O_19_ and SrFe_12_O_19_ single crystals [32–34], we found that the XRD patterns of the BaFe_12_O_19_ and SrFe_12_O_19_ single crystals are not consistent with that of magnetoplumbite-5H structure for M-type hexaferrites, i.e., the strong diffraction peaks from {110}, {007} and {114} lattice planes are absent. Those single crystals exhibited much higher symmetric structure than M-type hexaferrites. In addition, the single crystals were grown in a sealed furnace, which could result in heavy oxygen deficiency and induce the formation of large amountof oxygen vacancies and Fe^2+^ inside the crystals. Such crystals could producelarge current leakage during the electronic measurement and would appear a pseudo paraelectric phenomenon. Therefore the conclusion of quantum paraelectrics in those BaFe_12_O_19_ and SrFe_12_O_19_ single crystals [32–34] are not comparable with the ferroelectric behavior of PbFe_12_O_19_ specimen with magnetoplumbite structure. Actually, the doubtful ferroelectric property of SrFe_12_O_19_ ceramics with magnetoplumbite structure had already been reported several years ago in our previous study [35]; however, its ferroelectric hysteresis loops differ significantly from classic ferroelectric counterparts and resembled “bananas” due to the current leakage. Its ferroelectricityremains controversial and the banana-shaped P-E loops are not convincing evidence for its ferroelectricity [36]. The ME effect of SrFe_12_O_19_ has also not been investigated yet. Under this consideration,we optimized the fabrication process of the specimen by subsequent annealing SrFe_12_O_19_ specimen in oxygen atmosphere so as to remove the oxygen vacancies and transform Fe^2+^ into Fe^3+^. In this way, the current leakagewould be greatly reduced and a saturated P-E loop could appear as we did in PbFe_12_O_19_ specimens [29]. In this paper, we will then present the improved ferroelectric feature, enhanced impedance property, large dielectric anomaly near the Curie temperature, nonlinear I-V peaks, remarkable ME response, together with strong ferromagnetism in the M-type hexaferrite of SrFe_12_O_19_ ceramic specimen with subsequent O_2_ annealing process.

## Materials and Methods

We started with the preparation of nano-crystalline SrFe_12_O_19_ powders by polymer precursor procedure. Strontium acetate (Sr(CH_3_COO)_2_•3H_2_O) (99.0%, Aladin) and ferric acetylacetonate (C_15_H_21_FeO_6_)(99.9%, Alfa Aesar) were used as starting material. First of all, 0.2467g strontium acetatewas dissolved in 15 mL glycerin to form a clear solution. The solution was distilled in a rotary evaporator at 120°C for 1 h to remove the water trapped in Sr(CH_3_COO)_2_•3H_2_O. The distilled solution was transferred into a 50 mL flask, which was moved into a glove box. In order to avoid hydrolysis of the C_15_H_21_FeO_6_ compound in air, the following chemical process was carried out inside a glove box with argonatmosphere. 4.026 g of ferric acetylacetonate was weighed and dissolved in a mixture solution of 100 mL anhydrous ethanol and 70 mL acetone in a 250 mL three-neck flask inside the glove box. The solution was stirred at 70°C for 6 hours to ensure that ferric acetylacetonate was fully dissolved. Subsequently, the strontium and ferric precursor solutions were mixed together. Here, the molar ratio of strontium to iron was set to 1:9.5~10 to balance the Sr loss during the heat treatment process. Afterwards, 45 mL ammonia solution and 15 mL solution of polyethylene glycol were poured into the above mixture solution. The dispersion solution was maintained at 70°C under stirring for 24 hours and then moved out of the glove box. The water and organic molecules were removed by centrifuging the dispersion solutionat 12000 rpm for 30 minutes. The remaining colloid powders were calcined at 450°C for 1 hour. The powders were grinded in a agate mortar for 1 hour and then calcined again at 800°C for another hour to ensure total removal of organic molecules. In this way, pure SrFe_12_O_19_ powders in a single phase were obtained. 0.060 g of SrFe_12_O_19_ powders were weighed and pressed in a module into a pellet, which was then sintered at 1150°C for 1 hour into a solid ceramic specimen. The ceramic pellet was subsequently annealed in pure O_2_ at 800°C for 3 hours. Then the ceramic pellet was turned over with upside down and the annealing process was repeated again for another 3 hours. After the furnace was cooling down to room temperature, the ceramic was heat-treated in pure O_2_ once more at 700°C for 3 hours. In this way, the oxygen vacancies could be removed and Fe^2+^ would be fully transformed into Fe^3+^, so as to greatly enhance the resistance of the ceramics and reduce the current leakage during the following ferroelectric measurement. Phase identification of the SrFe_12_O_19_ powder and ceramic was performed by X-ray powder diffraction (XRD) with Cu–K_α_ radiation. Magnetization was measured using a physical property measurement system (PPMS). For dielectric and ferroelectric measurement, both surfaces of the ceramic pellets were coated with silver paste as electrodes which was heat treated at 820°C for 15 min; the P-E hysteresis loop was measured using a lab-constructed instrument, referred to ZT-IA ferroelectric measurement system. The temperature-dependent dielectric properties were measured by an LCR instrument (HP 8248A). The complex impedance spectrum was measured upon a electrochemical station (Chenghua) within the frequency range of 0.01Hz ~ 1MHz. The magnetocapacitance parameters of the SrFe_12_O_19_ pellet were measured using a Wayne Kerr 6500B LCR station by applying a variable magnetic field.

## Results and Discussion

### 1. Structure Identification of SrFe_12_O_19_ compound

**Fig 1:**
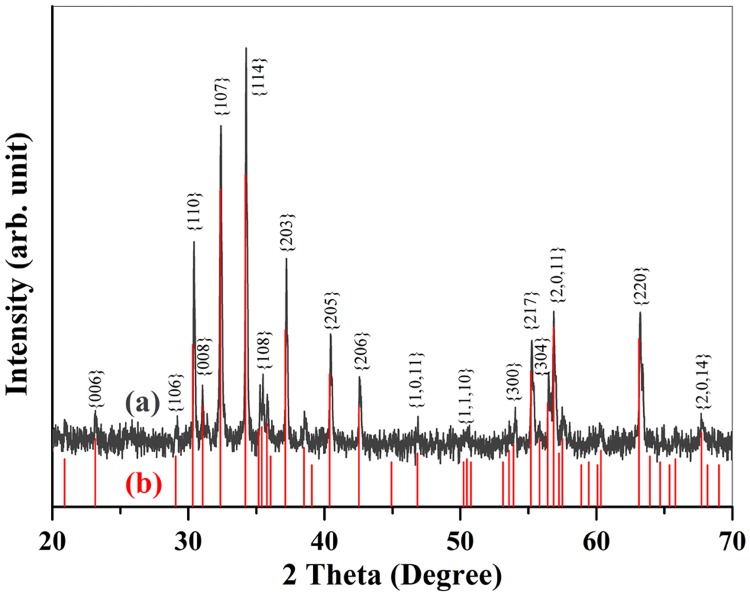
(a) XRD pattern of SrFe_12_O_19_ with O_2_ annealing process. (b) the standard diffraction pattern of the SrFe_12_O_19_(PDF#33–1340) being marked by discrete red lines.

Fig 1a shows the X-Ray diffraction (XRD) pattern of the as-preparedSrFe_12_O_19_ specimen, the underneath lines in red color are the standard diffraction spectrum of SrFe_12_O_19_ (PDF#33–1340). The single-phase SrFe_12_O_19_ powders has been fabricated by sintering at 1150°C for 1h and subsequently annealed in O_2_ by 3 steps for a total duration of 9 hours with 3 steps wise.

It can be seen from Fig 1 that all the diffractions peaks of the oxygen annealed specimen match well with the corresponding ones from the standard cards (*PDF#33–1340*), indicating the formation of pure SrFe_12_O_19_. No diffraction peaks from any second ferrite phases or impurity compounds have been indexed in the pattern, revealing the stability of the magnetoplumbite structure SrFe_12_O_19_ being sintered at 1150°C. This diffraction pattern is completely different from that of reported SrFe_12_O_19_ single crystals [32–34], which exhibit much higher symmetry and is lacking the strongest diffraction peaks form {110}, {007} and {114} lattice planes of typical M-type hexaferrites. Since the structure of our fabricated SrFe_12_O_19_ ceramics is different from those SrFe_12_O_19_ crystals reported in the literatures [32–34], the symmetry and electric properties should also differ significantly.

### 2. Electric Properties of SrFe_12_O_19_ Ceramics

In order to check out if oxygen annealing process could remove the oxygen vacancies and transform Fe^2+^ into Fe^3+^, we measured the complex impedance spectrum of the SrFe_12_O_19_ ceramics with and without O_2_ annealing by an electrochemical station. The annealing process was carried out in 3 steps, firstly the sintered ceramic was heat treated in O_2_ atmosphere in a sealed tube furnace at 800*°C* for 3 hours, then the specimen was turning over with upside down and once again annealed at the same temperature for another 3 hours, finally the annealed specimen was heat treated at 700*°C*for 3 hours. The complex impedance of PbFe_12_O_19_ can be expressed as follows:

$$\text{Z}={\text{Z}}^{\prime }+\text{j}{\text{Z}}^{"}=\frac{\text{R}}{1+{\left(\omega \text{RC}\right)}^{2}}-\text{j}\frac{\omega {\text{R}}^{2}\text{C}}{1+{\left(\omega \text{RC}\right)}^{2}}$$ (1)

and the module of the complex impedance is expressed as:

$$\left|\text{Z}\right|=\sqrt{\text{Z}\prime^{2}+\text{Z}{"}^{2}}$$ (2)

**Fig 2:**
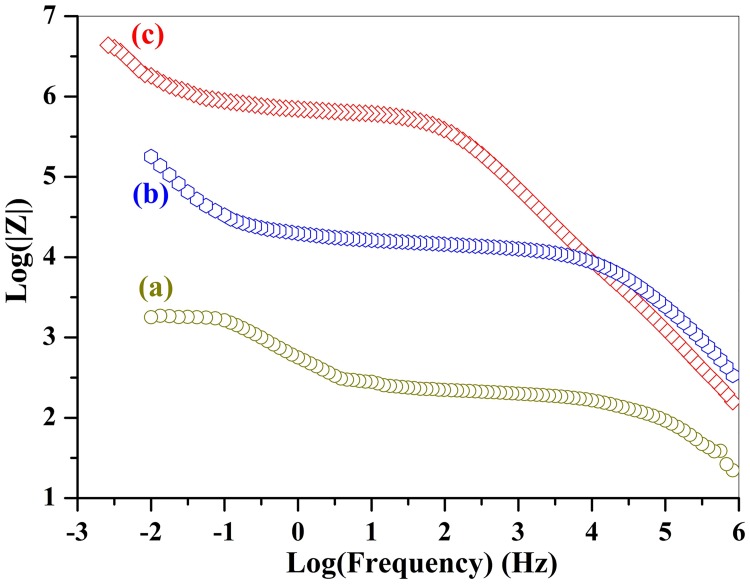
Modules of the complex impedance for (a) the conventional SrFe_12_O_19_ ceramics being sintered at 1150°C in air only, (b) the sintered ceramic was subsequently annealed in pure oxygen atmosphere at 800°Cfor 3 hours, (c) the annealed ceramic was flipping over and once again heat treated in O_2_at 800°C for another 3 hours.

Then we measured the complex impedance spectra of the specimens at each annealing step to show how the properties continually improve as the Fe^2+^ transforms to Fe^3+^. Fig 2 exhibits the modules of complex impedance for the SrFe_12_O_19_ ceramics without and with oxygen heat-treatment, respectively. The modules represents the magnitude of the impedance or electric resistance, which reflects concentration of oxygen vacancies and Fe^2+^ in SrFe_12_O_19_ ceramics. The higher is the module, the lower is the concentration of these charge carriers.

The impedance module or electric resistance of the specimen with O_2_ heat-treatment is much higher than that of the ceramic without O_2_ treatment within the whole frequency region. The electric resistance (impedance module) of the SrFe_12_O_19_ ceramic without O_2_ annealing process is only 1.8×10^3^ Ω, which is enhanced to 1.82×10^5^ Ω after first step O_2_ treatment. The electric resistance is further promoted to 7.9×10^6^ Ω at a frequency of 0.01 Hz after the second step O_2_ annealing process. The third step annealing process didn't change the module of impedance very much. The total electric resistance (impedance module) of the SrFe_12_O_19_ ceramic was enhanced by a factor of 4389 after annealing in O_2_ in 3 steps wise. The great enhancement of the resistance reveals the drastic reduction of the concentration of the oxygen vacancies and full conversion of Fe^2+^ into Fe^3+^, since the current leakage from oxygen vacancies and electronic hopping between Fe^2+^ and Fe^3+^ has been precluded.

**Fig 3:**
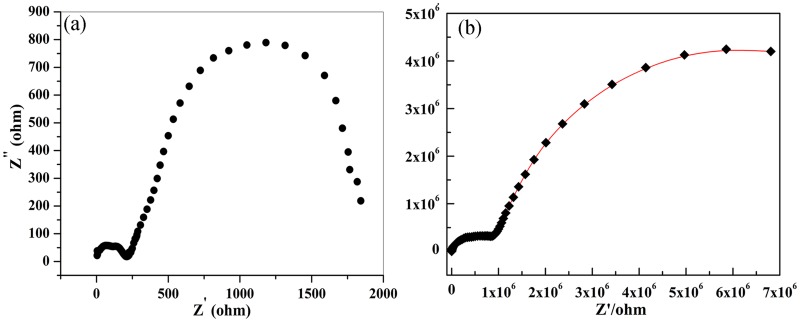
Complex impedance spectrum of the SrFe_12_O_19_ ceramic within the frequency range of 0.01 Hz to 1 MHz, (a) for the ceramic being sintered at 1150°C in air only; (b) for the sintered ceramic with subsequent annealing in O_2_.

**Fig 4:**
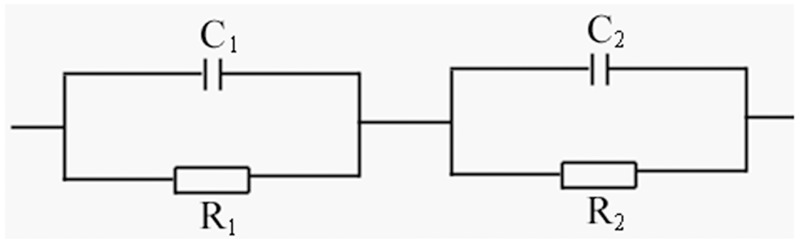
The equivalent circuit for the complex impedance of SrFe_12_O_19_ ceramics, the scheme is composed of two series linked sub-circuits with a capacitor and a resistor parallelly connected.

Fig 3a represents the complex impedance spectrum of SrFe_12_O_19_ ceramic without subsequent O_2_ annealing process. The spectrum is composed of a small Cole circle with a diameter of 215 and a large Cole one with a diameter of 1627. Each circle represents a circuit composed of a capacitor and a resistor which are connected in parallel. The two linked Cole circles could then be expressed as two such equivalent series connected circuits, as being shown in Fig 4. The small Cole circle contributes from the grain boundaries and the large one from the grains in SrFe_12_O_19_ ceramics. Fig 3(b) demonstrates a more complicated impedance spectrum for SrFe_12_O_19_ ceramics after O_2_ annealing process. The equivalent circuitfor the spectrum could also be expressed as two series linked circuits, each one is composed of a capacitor and a resistor being parallel connected (Fig 4). Each Cole circle corresponds to one individual circuit, one for grains and the other one for grain boundaries. Similarly, the spectrum is composed of a small Cole circle and a big half Cole circle, the diameter of the small one is estimated to be 9.0×10^5^ and that of large one is 9.8×10^6^. Obviously, the contribution of the impedance from both grain boundaries and grains in SrFe_12_O_19_ ceramics with subsequent O_2_ heat-treatment have been greatly enhanced in comparison with that without O_2_ annealing process. Both real and imaginary parts of the impedance have been promoted more than 1000 times after O_2_ heat-treatment.

### 3. The Impact of O_2_ Treatment on Charge Transfer by XPS

The heat treatment of the sintered SrFe_12_O_19_ ceramics in oxygen atmosphere plays a key role on enhancement of their electric resistance through removal of oxygen vacancies and transformation of Fe^2+^ into Fe^3+^. The change in concentration of these charge carriers could be detected by X-ray photoemission spectrum (XPS). We collected XPS data for two specimens, one is sintered SrFe_12_O_19_ ceramic without O_2_ heat treatment, the other one is the SrFe_12_O_19_ ceramic with subsequent O_2_ heat treatment at 3 steps wise. We did analysis on Fe 2p energy levels and shallow region of the valence bands for the two specimens.

**Fig 5:**
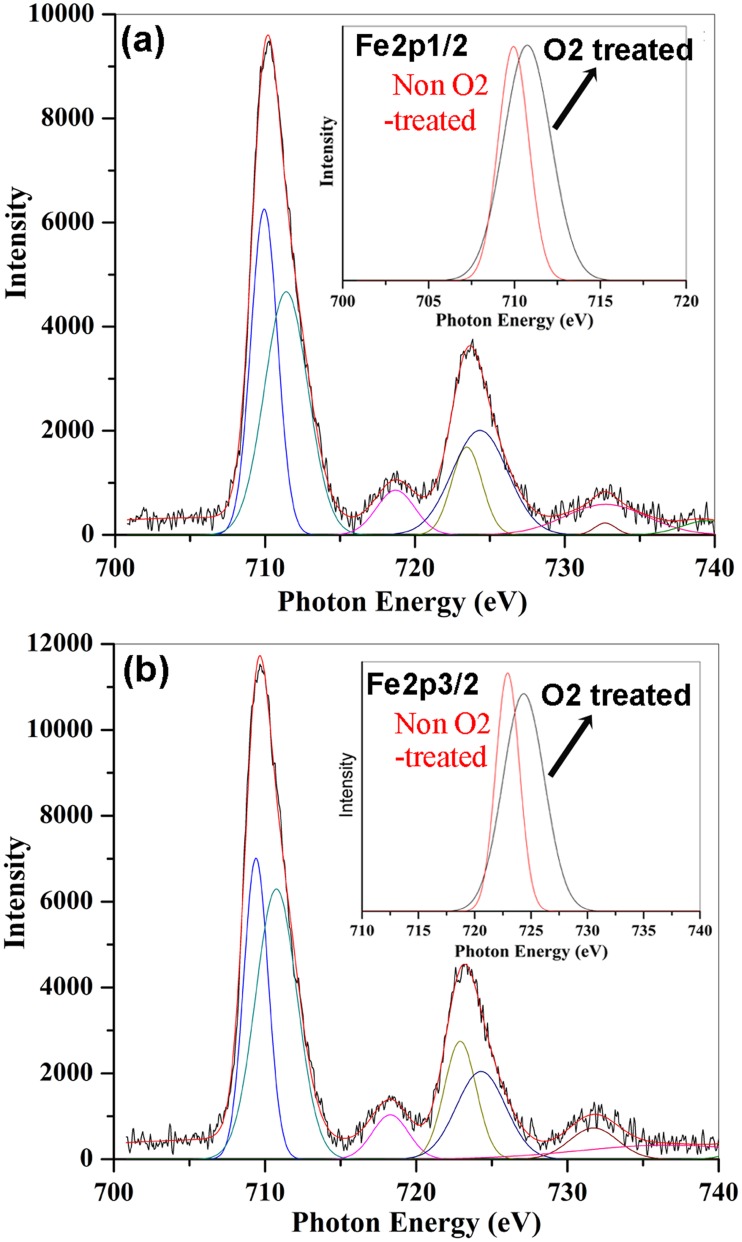
XPS spectrum for Fe 2p energy levels of the sintered SrFe_12_O_19_ ceramics (a) with O_2_ treatment and (b) without O_2_ treatment. The insets display the upper Fe2p1/2 and Fe 2p2/3 lines for the two specimens, respectively.

Fig 5 shows the spectra of Fe 2p energy levels for sintered SrFe_12_O_19_ ceramics with and without subsequent O_2_ annealing process. The Fe 2p3/2 peaks are centered at 710.4 eV and 709.68 eV for O_2_ treated specimen and non-O_2_ treated specimen, while that of Fe 2p1/2 peaks are positioned to 723.84 eV and 723.09 eV for O_2_ and non-O_2_ treated specimens, respectively. There appear chemical shifts of 0.72eV and 0.75 eV for Fe 2p3/2 and 2p1/2 lines between the two specimens, respectively. Both Fe 2p1/2 and 2p3/2 spectra are asymmetric and could be fitted into two symmetric peaks (Fig 5). The binding energies of the upper Fe 2p3/2 and 2p1/2 lines are fitted to be 711.41 eV and 710.5 eV, while that of lower fitting lines are positionedto 709.94 eV and 709.4 eV for O_2_ and non-O_2_ treated specimens, respectively. The chemical shifts of the fitting Fe 2p lines are displayed in the insets of Fig 5. Usually, the binding energies of 2p states of Fe^2+^ are lower than that of Fe^3+^. For example, the binding energy of Fe 2p3/2 in FeCl_3_ was measured to be 711.3 eV [37], while that in FeCl_2_ was 710.6 eV [37]. There was a chemical shift of 0.7 eV for the Fe 2p3/2 state between FeCl_3_ (Fe^3+^) andFe Cl_2_ (Fe^2+^). In our case, the binding energy of upper Fe 2p3/2 lines hifts from 710.5 eV to 711.41 eV after SrFe_12_O_19_ ceramic was heat treated in O_2_. The chemical shift of 0.91 eV appears between two specimens. The binding energy of upper Fe 2p3/2 line at 711.41 eV for O_2_ treated specimen is consistent with the value of Fe 2p3/2 line in FeCl_3_ (Fe^3+^) compound [37], indicating the existence of full Fe^3+^ in O-treated specimen. The lower binding energy of upper Fe 2p3/2 line at 710.5 eV indicates the existence of Fe^2+^ in non-O_2_ treated specimen. Both upper and lower Fe 2p3/2 and 2p1/2 states in non-O_2_ treated SrFe_12_O_19_ ceramic specimen indicates the existence of Fe^2+^ due to the chemical shifts. The binding energies of Fe 2p3/2 and 2p1/2 states shift toward higher energy side after the SrFe_12_O_19_ ceramics were subsequently annealed in O_2_ atmosphere, indicating the transformation of Fe^2+^ ions into Fe^3+^ ions with O_2_ treatment.

**Fig 6:**
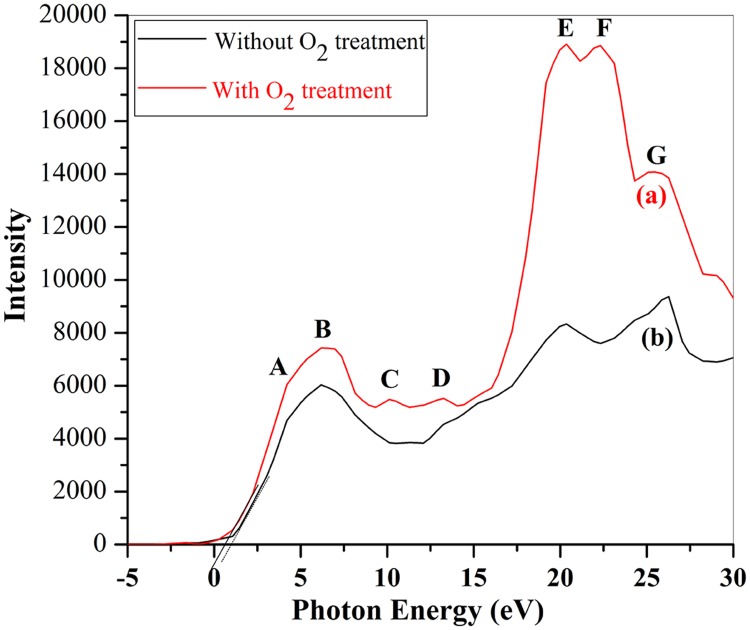
Valence band spectra of SrFe_12_O_19_ ceramics (a) with O_2_ heat treatment and (b) without O_2_ heat treatment. The dotted lines marked the top of the valence band.

Such charge transfer in Fe ions could be further confirmed by the Fe 3d states within the valence band region. Fig 6 shows valence band structure of SrFe_12_O_19_ ceramic with and without O_2_ treatment. There is large difference between the two spectra either in peak positions or the density of states. There are 7 peaks being marked by A, B,…,G in Fig 6. The binding energies and attribution of each peak are summarized in Table 1. Considering calculated electron density of states (DOS) of BiFeO_3_ and SeFe_12_O_19_ [38,39], we may assign both peaks A and B to the hybridized Fe 3d-O 2p states, peaks C and D to O 2p levels, peaks E, F and G to O 2s state which splits into 3 sub-energy levels. By comparing with the two valence band spectra, we could find that the density of hybridized Fe 3d-O 2p states (peaks A&B) has been enhanced after the specimen was O_2_ heat treated. The increment in density of Fe 3d state indicates more Fe^3+^ ions existing in the O_2_ treated specimen than that in non-O_2_ treated one, since Fe^3+^ions have one more unpaired 3d electron to be hybridized with O 2p electrons than Fe^2+^ ions. Both Fe 2p and 3d states in XPS spectra of SrFe_12_O_19_ ceramics confirmed the transformation of Fe^2+^ into Fe^3+^ after the ceramics were subsequently annealed in pure O_2_ atmosphere.

**Table 1:** Valence band states assignment.

Peak	A	B	C	D	E	F	G
Binding Energy	4.18(eV)	6.22(eV)	10.06(eV)	13.27(eV)	20.32(eV)	22.37(eV)	25.73(eV)
Assignment	Fe3d+O2p	Fe3d+O2p	O 2p	O 2p	O 2s	O 2s	O 2s

It can be seen from Fig 6 that there appear full O 2p and 2s states in O_2_-treated SrFe_12_O_19_ ceramic, while two O 2p states of peaks C and D are absent in non-O_2_ treated SrFe_12_O_19_ ceramic. The density of O 2s states (peaks E, F and G) in O_2_-treated specimen are much higher than that of SrFe_12_O_19_ ceramic without O_2_ heat treatment, the intensity of peaks E and F increases from around 8306 (Fig 6b) for the non-O_2_ treated specimen to around 18922 (Fig 6a) for the O_2_ treated specimen. The great enhancement of density of O 2s and O 2p states indicates largely reduction of oxygen vacancies for the SrFe_12_O_19_ ceramics after O_2_ heat treatment. When the SrFe_12_O_19_ pellet was sintered inside a sealed furnace into ceramics, large numbers of Fe^2+^ would be formed and the excess oxygen would exist in the form of oxygen vacancies due to oxygen deficiency. The oxygen vacancies, however, are positively charged only and have no such 2s and 2p electrons as those around the atomic nucleus in oxygen ions. Therefore the density of O 2s and 2p states are much more depressed in the valence spectrum (Fig 6b) of non-O_2_ treated SrFe_12_O_19_ ceramics due to the appearance of high concentration of oxygen vacancies. The higher is the content of oxygen vacancies, the lower is the valence electron density. After the SrFe_12_O_19_ ceramics are subsequently heat treated in pure O_2_ atmosphere, Fe^2+^ ions are oxidized to Fe^3+^ and oxygen vacancies could be greatly reduced and be replaced by oxygen ions, as such the density of O 2s and 2p states are greatly improved. Therefore, the appearance of additional two O 2p states (peaks C and D) as well as the great enhancement of the density of O 2s states confirm the removal or great reduction of oxygen vacancies after the specimen was annealed in O_2_ atmosphere. This result is consistent with the enhancement of electric resistance after the specimen was heat treated in O_2_. Meanwhile, the valence band edge of SrFe_12_O_19_ shifts upwards 0.39 eV after it was annealed in O_2_ atmosphere.

### 4. Dielectric Relaxation of O_2_-Treated SrFe_12_O_19_ Ceramics

**Fig 7:**
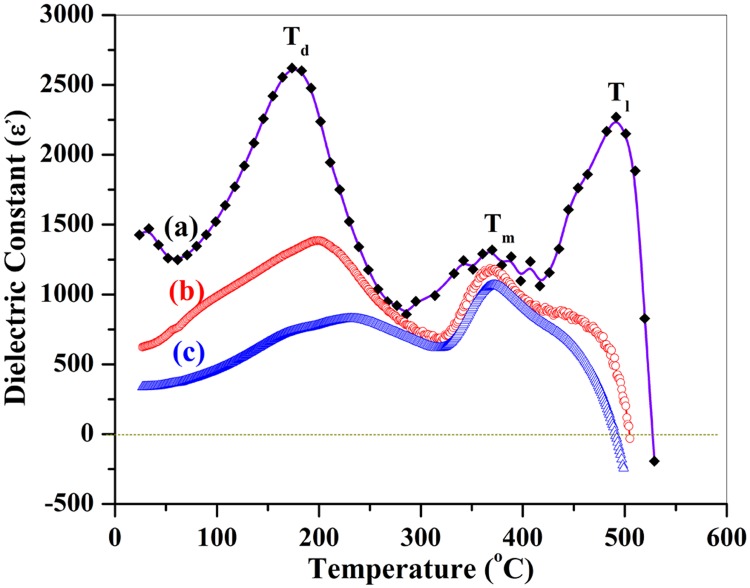
Plot of dielectric constant as a function of temperature for SrFe_12_O_19_ ceramics with O_2_ treatment at frequencies of (a) 1kHz, (b) 10kHz and (c) 100 kHz.

We then measured the dielectric relaxation behavior of O_2_ treated SrFe_12_O_19_ ceramicsby a HP4284A LCR instrument. Fig 7 shows the temperature-dependent dielectric constants of the specimen at different frequencies of 1kHz, 10kHz and 100 kHz. At 1kHz, there appear three peaks, locating at 174°C, 368°C and 490°C (Fig 7a), the first two peaks are similar to that of PbFe_12_O_19_ corresponding to two kinds of phase-transitions [29]. Similarly, the first peak T_d_ could be assigned to the ferroelectric to anti-ferroelectric phase transition, while the second one (T_m_) to the anti-ferroelectric to para-electric phase transition. The maximum dielectric constant at T_d_ is 2621. The third peak is attributed from a complicated phase transition. Since the dielectric constant (ε) becomes negative when temperature is higher than 527°C (Fig 7), the phase structure could then be assigned to a so-called "left hand materials (LHM)" whose dielectric constant is less than 0. Therefore the third peak (T_l_ = 527°C) is proposed to be the phase transition from para-electric phase to LHM.

The first transition peak is sensitive to the frequency, the larger is the frequency, the higher is the transition temperature. When the frequency increases from 1 kHz to 10 kHz, the first transition peak shifts from 174°C to 199°C and the maximum dielectric constant drops from 2261 to 1394. Further increasing the frequency from 10 kHz to 100 kHz, this peak shifts to 239°C and the maximum dielectric constant decreases from 1394 to 847. However, the second transition peaks didn't move accordingly, while the third peak (T_l_) moves to the opposite direction, the higher is the frequency, the lower is the transition temperature.

The temperature of the first dielectric constant peak show large shifts with frequencies, suggesting that SrFe_12_O_19_ is a relaxor ferroelectric compound with a diffuse phase transition. At 1kHz, the maximum ε-T peak (490°C) demonstrates a strong ferroelectric to antiferroelectric phase transition, associated with a broad ε (*T*) anomaly near the vicinity of the transition temperature. These kinds of dielectric anomalies at different frequencies provides additional evidence for the ferroelectricity of SrFe_12_O_19_.

Upon the anomalies of the dielectric constants, we then made the calculation of the reciprocal dielectric constants as a function of temperature using modified Curie-Weiss law, being expressed as follows:

$$\frac{\text{1}}{\varepsilon }\text{-}\frac{\text{1}}{\varepsilon_{\text{m}}}=C{\left(T-T_{m}\right)}^{\gamma }$$ (3)

where γ is the critical exponent, representing the degree of diffuseness of the transition, and *C* is a Curie–Weiss-like constant. ε_m_ is the maximum dielectric constant at temperature of transition peak T_m_. For a sharp transition, γ = 1, the materials are called normal ferroelectrics. Diffuse transitions lie in the range 1 <γ< 2 [40], whereas at γ = 2 the materials correspond to a so-called “complete” diffuse phase. When γ> 2, the materials would take a diffuse phase transition from ferroelectrics to anti-ferroelectrics or antiferroelectrics to paraelectricity [29].

**Fig 8:**
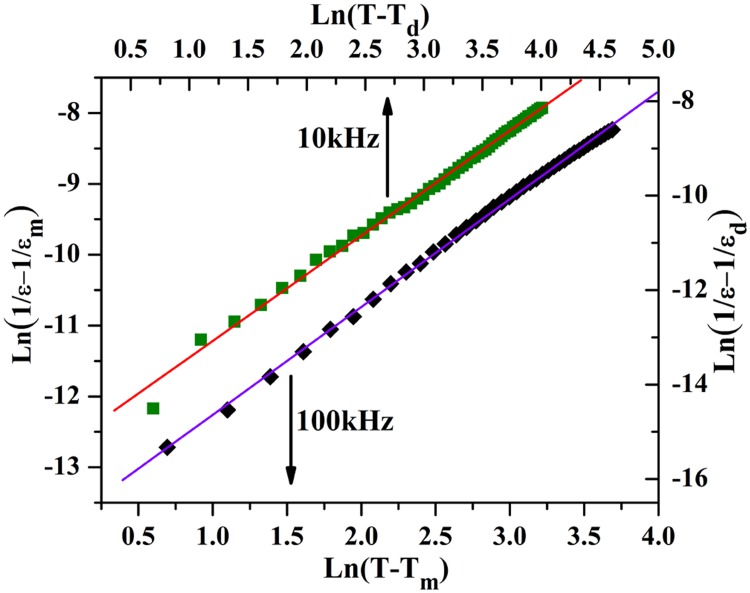
Modified Curie-Weiss law calculation. (a) Logarithm of (1/ε-1/ε_d_) as a function of logarithm of (T-T_d_) at 10 kHz. and (b) logarithm of (1/ε-1/ε_m_) as a function of logarithm of (T-T_m_) at 100 kHz for the SrFe_12_O_19_ ceramic being sintered at 1150°C for 1 hour and subsequently annealed in O_2_ for 9 hours with 3 steps wise.

Fig 8 shows plots of Ln(1/ε-1/ε_d_) as a function of Ln(T-T_d_) at 10 kHz and Ln(1/ε-1/ε_m_) as a function of Ln(T-T_m_) at 100kHz for SrFe_12_O_19_ ceramic, respectively. Linear fitting to the experimental datausing Curie-Weissformula derives out the slope of the fitting lines, which were determined to be γ = 2.3 and 2.2 at frequencies of 10 KHz and 100kHz, respectively. The calculated lines following Curie-Weiss formula fit well with the experimental data points. The linear relationship between Ln(1/ε-1/ε_m_) and Ln(T-T_m_) reveals that the temperature dependence of the dielectric constant obeys the Curie–Weiss law, providing additional evidence for the relaxor ferroelectric feature of the SrFe_12_O_19_ ceramics.

### 5. Ferroelectric Polarization of SrFe_12_O_19_ Ceramics

The ferroelectric P-E loop of the SrFe_12_O_19_ ceramics without O_2_ heat-treatment was looking like a "banana" (Figure E in S1 File) [35], which had drawn lots of doubts on the validity of its ferroelectricity. Considering that the "banana" shaped P-E loop (Figure E in S1 File) could be induced by current leakage and the necessity of confirming the validity of its ferroelectricity, we then heat treated the SrFe_12_O_19_ ceramics in pure oxygen atmosphere for total duration of 9 hours with 3 steps wise, so as to greatly enhance its resistance through reducing the concentration of charge carriers. The great reduction of the concentration of charge carriers, such as oxygen vacancies and Fe^2+^ by annealing the ceramics in oxygen,could dramatically reduce the current leakage and thus saturate the ferroelectric hysteresis loop of the SrFe_12_O_19_ ceramics.

**Fig 9:**
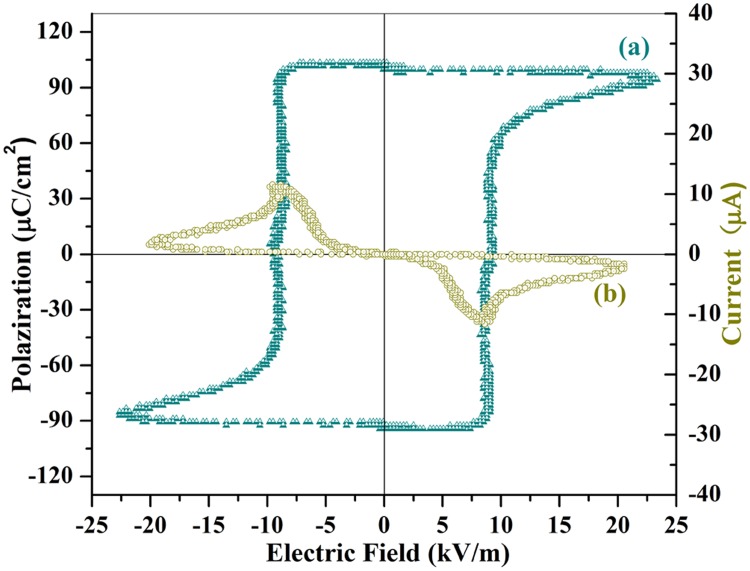
(a) The saturated ferroelectric polarization hysteresis (P-E) loop, and (b) a plot of current as a function of voltage (I-V curve) of SrFe_12_O_19_ ceramic. The ceramic has been sintered at 1150°C for 1 hour and subsequently annealed at 800°C in pure oxygen for a total duration of 9 hours in 3 steps wise. The measurement was made at a frequency of 33Hz and room temperature (300K).

Fig 9 shows a fully saturated ferroelectric hysteresis (P-E) loop of the SrFe_12_O_19_ ceramic with O_2_ annealing process. A drastic variation of the polarization appears in the vicinity of the specimen’s coercive field at around 10 kV/m. Further increasing the applied field up to 25 kV/m, the polarization of the ceramic gradually approaches to saturation along with a concave arc line (Fig 9a).

When the applied field decreases, the polarization remains at the value of saturation because most of the ceramic’s domains still align themselves along the electric field’s direction. The spontaneous polarization, which is equal to the saturation value of the electric displacement extrapolated to the zero-field strength, remains almost constant with external field variations. This result reveals that all the electric displacement dipoles have aligned themselves along the direction of the external field until the external field was less than the negative coercive field of the ceramics. When the applied field switched to the reversal direction, the spontaneous polarization demonstrated a hysteresis and changedthe direction suddenly at the position of negative coercive field. The polarization voltage changes signs to be negative and approaches to the negative saturated value along with a reversal concave arc line (Fig 9a) within the field range of -10 kV/m to -25 kV/m. The remnant polarization in this classic hysteresis loop is estimated to be103μC/cm^2^, which is around 8.3 times higher than that (15μC/cm^2^) of SrFe_12_O_19_ ceramics without subsequent heat treatment in O_2_ [35]. Therefore subsequent annealing SrFe_12_O_19_ ceramics in oxygen not only saturated the hysteresis loop, but also greatly improved the remnant polarization value through reducing the current leakage, which results from the removal of oxygen vacancies and the transformation of Fe^2+^ to Fe^3+^ [28, 29]. Similar ferroelectric hysteresis loops being measured on different SrFe_12_O_19_ ceramic specimens are supplied in the S1 File (Supplementary Materials), so as to confirm the reliability and repetitiveness of ferroelectric data.

The last evidence for the validity of ferroelectricity for SrFe_12_O_19_ ceramics would be attributed from the appearance of two current peaks at I-V plot (Fig 9b) along with the polarization switching. When the ferroelectric polarization is switching, the screening surface charges flow from one electrode to the other one and create momentarily a sudden change of current. In the current versus voltage plot, this will result in two peaks with reversal directions as being shown in Fig 9(b). The two nonlinear I-V peaks show very clearly the switching phenomenon of the polarization and does not present any linear current component (or current leakage component). The two I-V peaks are similar to that of typical ferroelectric compounds (Pb(Zr_0.4_Ti_0.6_)O_3_and LiNbO_3_) [41] and could convince us that the P-E hysteresis loop indeed origins from the ferroelectric polarization instead of current leakage. The origin of the ferroelectricity of SrFe_12_O_19_ ceramics is similar to that of PbFe_12_O_19_ and has been discussed in detail in our previous literatures [28, 29], since both compounds share the same crystal structure. The off-center shift of the Fe^3+^ ions and the displacement of O^2-^ ions from its original corner positions in the FeO_6_ octahedron are supposed to be the origin of electric polarization in SrFe_12_O_19_ too [28, 29]. The saturated ferroelectric hysteresis loop, two peaks in the I-V curve, the giant anomalies of the dielectric constant in the vicinity of the transition temperatures (T_m_ and T_d_) as well as the comply of the reciprocal dielectric constant with modified Curie Weiss law provide us with enough evidences to prove the intrinsic ferroelectricity of SrFe_12_O_19_ ceramics.

This result is quite different from that reported M-type hexaferrtie single crystals, which were claimed to be a new family of magnetic quantum paraelectrics and retained paraelectric symmetry down to zero temperature [32–34]. Actually those crystals showed different crystal structure with higher symmetry and were grown in a sealed furnace without subsequent heat-treatment in oxygen atmosphere. There would be high concentration of oxygen vacancies and Fe^2+^ ions inside the crystals. These kinds of carrier charges would reduce the electric resistance of the crystals and induce large current leakage during the electric measurement. As such no saturated polarization hysteresis loop could be observed in these crystals. Both different crystal structure and low concentration of carrier charges make our ceramic specimens differ significantly from those crystals in ferroelectric and dielectric properties.

### 6. Magnetic Properties of SrFe_12_O_19_ Compound

**Fig 10:**
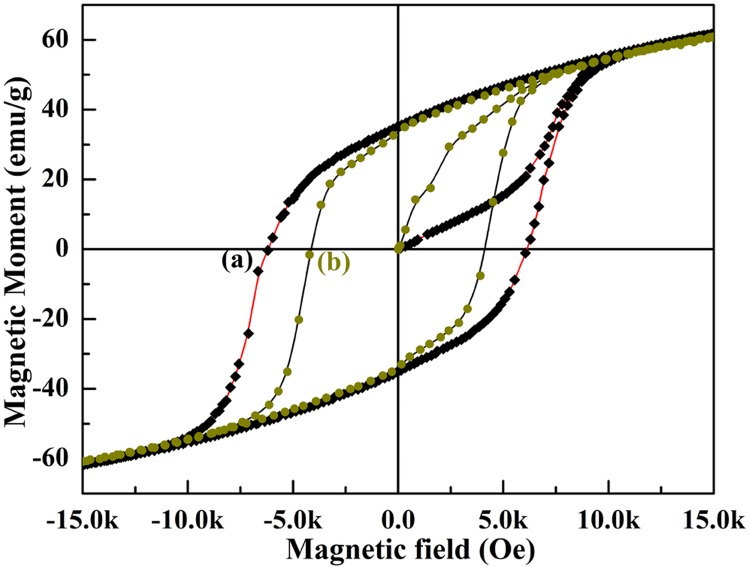
Magnetic hysteresis loop of SrFe_12_O_19_ (a) being sintered at 1150°C for 1 h and subsequently annealed in O_2_ for 9 hs with 3 steps wise, (b) without heat-treatment in O_2_.

For magnetic measurement, the SrFe_12_O_19_ powders were prepared with the same heat-treatment history as that of above ceramics. The magnetic measurement was made upon SrFe_12_O_19_ powders by the Physical Property Measurement System (PPMS) at room temperature. Fig 10 exhibits the ferromagnetic hysteresis loops of SrFe_12_O_19_ powders with and without O_2_ heat-treatment. It can be seen that magnetic properties of SrFe_12_O_19_ have been greatly improved by annealing the powders in oxygen atmosphere. The coercive fields of the SrFe_12_O_19_ powders with O_2_ treatment reaches as high as 6192 Oe, while that of the same powders without O_2_ treatment is 4111 Oe. The coercive field has been promoted 2081 Oe through O_2_ heat-treatment. The remnant magnetic moment has also been enhanced from 33.5 emu/g to 35.8 emu/g after annealing the SrFe_12_O_19_ powders in oxygen atmosphere. The increase in remnant magnetization is quite modest since the applied magnetic field is not high enough to have all of the domains align themselves parallelly to the external field. Such promotion of magnetic polarization was also observed in M-type lead hexaferrite (PbFe_12_O_19_) after annealing in O_2_ atmosphere [29].

The heat-treatment in oxygen atmosphere transformed Fe^2+^ into Fe^3+^, which provides one more unpaired electron spin for magnetic polarization of SrFe_12_O_19_. The existence of more Fe^3+^ in SrFe_12_O_19_ would promote its magnetic properties. On the other hand, a certain content of Fe^2+^ existing in SrFe_12_O_19_ which results from sintering the ceramics in a oxygen deficient atmosphere, such as a sealed air furnace, would reduce its ability for magnetic polarization and degrade its magnetic properties since Fe^2+^ contains one less electron spins than Fe^3+^.

The evidence for increment of Fe^3+^ content in SrFe_12_O_19_ ceramics after O_2_ treatment is the great promotion of the coercive magnetic field in the M-H loop of Fig 10. The coercive field is the intensity of the applied magnetic field required to reduce the magnetization of that material to zero after the magnetization of the sample has been driven to saturation. This value reflects the ability of spontaneous magnetic polarization of the magnetic materials. All the magnetic dipoles or spins are aligned anti-parallelly to the external magnetic field at the coercive point, whose value equals to the full magnetization in the opposite direction to withstand an external magnetic field without becoming demagnetization. The more is the content of Fe^3+^ in SrFe_12_O_19_ compound, the higher is the concentration of unpaired electron spins and thus the larger is the ability of its full spontaneous magnetic polarization, which would need higher external magnetic field to balance the anti-parallelly aligned magnetic dipoles or spins. Therefore the coercive field would be promoted if there are more Fe^3+^ ions than Fe^2+^ ions in SrFe_12_O_19_ compound. When the dipoles or spins are all anti-parallelly aligned to the external field at the coercive point of -4154 Oe with 0 magnetization in SrFe_12_O_19_ ceramics without O_2_ treatment (Fig 10a), there are still many parallelly aligned dipoles or spins in the O_2_ treated SrFe_12_O_19_ ceramics, whose net magnetization still remains at 21.05 emu/g at this point (Fig 10b & Figure A in S1 File). By comparing the increase of Bohr magnetrons at this field point (-4154 Oe) between the two samples, we are able to estimate the number of Fe^2+^ that converts to Fe^3+^.

Under this consideration, we calculated the molar susceptilbility from the measured B-H datasets of both samples through the equation:

$$\chi_{\;m}=\chi M/w$$ (4)

where *χ* = *B*/*H* (B = magnetic moment in emu, H = external field in Oe), M is the molar mass of SrFe_12_O_19_, *w* is the weight of the sample. Afterwards, the molar susceptilbility is converted to molecule magnetic moments by the equation:

$$\mu_{m}=\sqrt{3\chi_{m}kT/L\mu_{0}}$$ (5)

where L = 6.022×10^23^ mol^-1^ (Avogadro's number), k = 1.380×10^−23^ J·K^-1^ (Boltzmann constant), *μ*_0_ = 4π×10^-7^N·A^-2^ (vacuum permeability), T = temperature. The field dependent molecule magnetic moments are displayed in the (Figure A in S1 File). Upon the value of molecule magnetic moments, the unpaired electron numbers could then be calculated through the equation of $\mu_{B}=\mu_{m}\sqrt{n(n+2)}$, where *μ*_*B*_ = *eh*/2*m* = 9.274 × 10^−24^ *J*·*T*^−1^ (Bohr magnetron, h is the Planck constant), n is the number of unpaired electrons, *μ*_*m*_ is molecule magnetic moments. In this way, *μ*_*m*_ is calculated to be 0.9285×10^−23^ J·T^-1^ and 3.3759 ×10^−23^ J·T^-1^ for the non-O_2_ treated and O_2_ treated SrFe_12_O_19_ ceramics at point of -4154 Oe, which is the coercive field of the non-O_2_ treated sample (Fig 10a & Figure A in S1 File). Finally, the number of unpaired electrons is determined to be n_1_ = 0.414 and n_2_ = 2.772 for the non-O_2_ treated and O_2_ treated SrFe_12_O_19_ ceramics, respectively. Therefore, the difference of the unpaired electron numbers between two samples is Δ*n* = *n*_2_ –*n*_1_ = 2.358. At this point of view, The second sample (O_2_ treated SrFe_12_O_19_ ceramic) has around 2.4 more unpaired electrons than the first one (non-O_2_ treated SrFe_12_O_19_ ceramic), indicating that around 2.4 Fe^2+^ ions convert to Fe^3+^ ions after the SrFe_12_O_19_ ceramic was heat treated in O_2_atmosphere.

Therefore heat-treatment of SrFe_12_O_19_ in oxygen would not only improve the ferroelectric polarization performance but could also enhance the ferromagnetic properties through transforming Fe^2+^ into Fe^3+^. The large hysteresis loop reflects the strong magnetic feature of SrFe_12_O_19_. The above combined results demonstrate the simultaneous occurrence of large ferroelectricity and strong ferromagnetism in the single SrFe_12_O_19_ compound at room temperature. It allows us to expect a new generation of electronic devices being made of such a practicable multiferroic candidate, in which large ferroelectricity and strong ferromagnetism coexist.

### 7. Magnetocapacitance Effect of SrFe_12_O_19_ Ceramics

**Fig 11:**
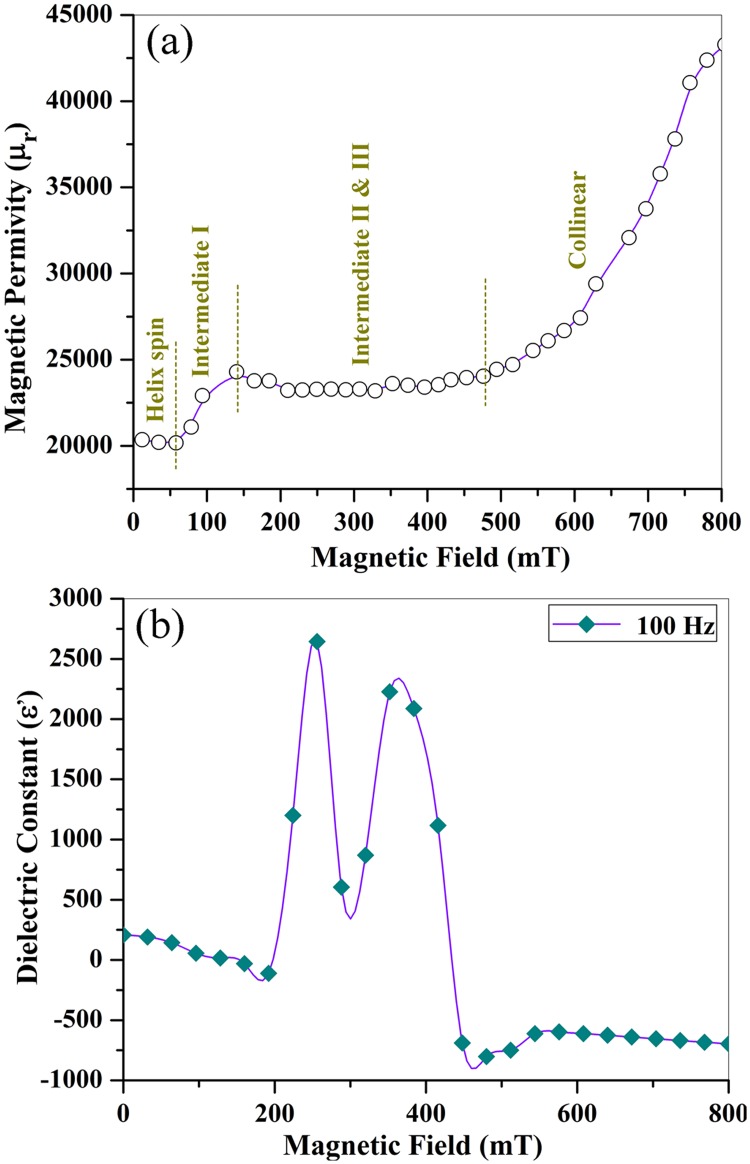
Giant magnetocapacitance effect: (a) B-dependent magnetic permeability (μ_r_) and delimited magnetic phase profile; (b) variable dielectric constant as a function of B field for the O_2_ treated SrFe_12_O_19_ ceramics at a frequency of 100Hz and room temperature (300K).

Previous studies on certain rare-earth manganites [6, 42] suggested that materials having long wavelength magnetic structures often exhibit a strong interplay between magnetic ordering and ferroelectricity, which makes the capacitance of the manganites [43] and Y-type hexaferrites exhibit great response to the B field [44]. In order to check out if the M-type strontium hexaferrite (SrFe_12_O_19_) could also generate such coupling response upon an external magnetic field, we set up a simple system for the ME coupling measurement, which was performed by measuring the capacitance as a function of the magnetic field (B). The SrFe_12_O_19_ ceramic was coated with silver electrodes on both sides and then placed in a space between two electromagnets. Upon the application of the magnetic field, the Wayne Kerr 6500B LCR Precision impedance analyzer, which was linked with the electrodes on both surfaces of the ceramic, would output the variable capacitance with the external magnetic field B. The B-field-dependent relative magnetic permeability was calculated using a defined formula, which can be expressed as follows [45]:

$$\mu_{r}=\frac{Z(T)-Z(0)}{if\mu_{0}h\text{ln}\frac{c}{b}}+1=[\frac{Z\prime \prime (T)-Z\prime \prime (0)}{f\mu_{0}h\text{ln}\frac{c}{b}}+1]-i[\frac{Z\prime (T)-Z\prime (0)}{f\mu_{0}h\text{ln}\frac{c}{b}}]$$ (6)

where Z(T) is the complex impedance when magnetic field B = T, Z(0) the impedance for B = 0; *μ*_0_ is the vacuum permeability, h is the space between two magnets, c and b are the inner and outer radius of the ring magnets. Fig 11 displays the dependence of the relative magnetic permeability (*μ*_*r*_) on B and the change in ε (or magnetocapacitance) along with B field. It can be seen from Fig 11a that *μ*_*r*_ increases in a stepwise fashion, which is attributed to the evolution in magnetic structures. Five successive magnetoelectric phases could be thus mapped out: the first terrace (0<B<55mT) for modified helix, the ramp (55mT<B<150mT) for intermediate I, the second terrace (140mT<B<480mT) for intermediate II and III. The collinear ferrimagnetic phase is assigned to the region (480mT<B<883mT) with a rather large slope in *μ*_*r*_. These magnetic phases has been delimited by yellow dot lines (Fig 11a).

Fig 11b displays great response of the capacity (or ε) to the applied magnetic field (B). The dramatic variation of dielectric constant with B field is similar to that of M-type BaFe_12-x_Sc_x_O_19_ [42] and Y-type hexaferrite Ba_0.5_Sr_1.5_Zn_2_(Fe_1-x_Al_x_)_12_O_22_ [20], and is associated with the magnetic phase transition along with B field. Within helix and intermediate I phases in range of 0~150 mT, the dielectric constant displays a downward slope line with small declining value (from 208.2 to -134.5). However, when B comes into the Intermediate II phase, the dielectric constant increases rapidly from -134.5 to 2650. The great change in ε is expressed as two remarkable peak structures centered in the middle of Intermediate II phase (254 mT) and III phase (363 mT), respectively (Fig 11b). The maximum dielectric constants of the two peaks are determined to be 2650 and 2340, respectively. The dielectric constants show rapid drops at the magnetic boundary between Intermediate II and III (254 mT<B<300mT) and that between Intermediate III and collinear phases (300 mT<B<48 mT). The valley bottom (ε = 66) between the two peaks locates at 300 mT, which could be assigned to the borderline between intermediate II and III magnetic phases.

When B comes across into ferrimagnetic collinear phase (B>480), the dielectric constant remains almost as a flat line (ε~-730) with little fluctuation, whose amplitude is less than 90. The maximum relative change in ε (Δε(B)/ε(0) = [ε(B)-ε(0)]/ε(0), where B = 254mT) is 1174%, which reflects a giant magnetocapacitance effect of SrFe_12_O_19_ ceramics. This magnetocapacitance order is much higher than that of YMnO_3_ thin films with a magnitude of only 5.5% at 3T [46]. Thus, the hexaferrite with long-wavelength magnetic structures exhibits remarkable ME responses at low B fieldand roomtemperature, which opens substantial possibilities for applications of ME systems.

The magnetic phase within the B region of 450mT<B<800mT could also referred to the so called "left hand materials" with negative capacitance (dielectric constants). Although negative capacitance is known to exist in semiconductors [47] and ferroelectrics, where it has been predicted theoretically [48], its direct measurement has been elusive so far [49]. Sayeef Salahuddin and colleagues has reported that negative capacitance in ferroelectrics can be directly measured by putting a large resistance between the voltage supply and the electrodes of the ferroelectric capacitor [50], such a negative capacitance is just a transient phenomenon and is intrinsically unstable because it exists only around the tipping point between the two thermodynamically stable polarization states [49, 50]. This complicates practical implementation. Here, we happened to observe the negative capacitance (or dielectric constant) in O_2_ treated SrFe_12_O_19_ ceramicsbeing stimulated by low magnetic field. The negative capacitance could remain stable under application of low B field (450mT<B<800mT). Such low B-field induced negative capacitance was also experimentally measured in another M-type Hexaferrite (La_0.2_Pb_0.7_Fe_12_O_19_), whose lowest dielectric constant reaches as small as -84866.2 at the B field of 700 mT [51]. The negative capacitance of ferroelectrics could offer a solution to a bottleneck in transistor miniaturization: transistors are becoming too small and clock-speed too fast to remove the heat generated during switching, resulting in increased power dissipation and over heating [49]. Amplification of low gate voltage using negative capacitance would enable low-power operation and could overcome such bottleneck problem in transistor miniaturization process.

In brief, large ferroelectricity and strong ferromagnetism are naturally merged together in one single SrFe_12_O_19_ compound, due to the coexistence of the off-centered FeO_6_ octahedron in its sub-unit cell and electron spins in partially filled 3d orbits of the Fe^3+^ ions. Thus, the mutually exclusive electric and magnetic orders are naturally integrated in one single SrFe_12_O_19_ compound. Therefore, large ferroelectricity, strong ferromagnetism and giant ME coupling effect are all synchronously realized in one single phase of SrFe_12_O_19_ at room temperature (300K).

## Conclusion

In summary, our work directly demonstrates the coexistence of large ferroelectricity and strong ferromagnetism in M-type strontium hexaferrite (SrFe_12_O_19_) at room- temperature (300K). We not only merge together the electric and magnetic orders, but also realize the giant magnetocapacitance effect in one single SrFe_12_O_19_ compound at room temperature. The SrFe_12_O_19_ ceramic displays a classical polarization hysteresis loop (P-E) with full saturation, two particular nonlinear I–V peaks, and dielectric anomalies near the Curie temperature, all of which verify its intrinsic ferroelectricity. Subsequent annealing the SrFe_12_O_19_ ceramic in oxygen atmosphere greatly enhance its electric resistance through removal of oxygen vacancies and transformation of Fe^2+^ into Fe^3+^, leading to the full saturation of the P-E loops. XPS spectra revealed the experimental evidences for great reduction of oxygen vacancies and transformation of Fe^2+^ into Fe^3+^ in SrFe_12_O_19_ ceramics upon annealing in O_2_. The remnant polarization of the SrFe_12_O_19_ ceramics is 103μC/cm^2^. Large magnetic hysteresis loop was also observed in SrFe_12_O_19_ due to its strong ferromagnetism. Furthermore, the capacitance (or dielectric constant) exhibits dramatic variation along with B field. The magnetic structure profile has been mapped out upon the relative magnetic permeability. Two remarkable peak structures of *ε*appeared at the centers of Intermediate II and III magnetic phases, respectively. The maximum relative change in *ε* is 1174%, which reflects a giant magnetocapacitance effect of SrFe_12_O_19_ ceramics. Low B-field induced negative capacitance was also observed in this compound.

## Supporting Information

S1 File SFO19 Supplementary File. (DOCX)

## References

[1] Zheng R. Y., Wang J., Ramakrishna S., J. Appl. Phys. 2008, 104, 0341061.

[2] Cheong S. W., Mostovoy M. V., Nat. Mater. 2007, 6, 13 10.1038/nmat1804 17199121

[3] Khomskii D. I., Physics 2009, 2, 20

[4] Lottermoser T., Lonkai T., Amann U., Hohlwein D., Ihringer J., Fiebig M., Nature 2004, 430, 541,. 10.1038/nature02728 15282600

[5] Carlos A. F. V., Hoffman J., Ahn C. H., Ramesh R., Adv. Mater. 2010, 22, 2900 10.1002/adma.200904326 20414887

[6] Tan G. L., Chen X. N., Magnetism J. and Magnetic Materials, 2013, 327, 87.

[7] McKee R. A., Walker F. J., Chisholm M. F., Phys. Rev. Lett. 1998, 81, 3014.

[8] Reiner J. W., Walker F. J., Ahn C. H., Science 2009, 323, 1018 10.1126/science.1169058 19229025

[9] Levin I., Li J., Slutsker J., Roytburd A. L., Adv. Mater. 2006, 18, 2044.

[10] Hill N. A., Annu. Rev. Mater. Sci. 2002, 32, 1.

[11] Eerenstein W., Mathur N. D., Scott J. F., Nature (London) 2006, 442, 759. 16915279 10.1038/nature05023

[12] Tokura Y., Science 2006, 312, 1481 10.1126/science.1125227 16763137

[13] Yamasaki Y., Miyasaka S., Kaneko Y., He J. P., Arima T., Tokura Y., Phys. Rev. Lett. 2006, 96, 207204 10.1103/PhysRevLett.96.207204 16803202

[14] Katsura H., Nagaosa N., Balatsky A. V., Phys. Rev. Lett. 2005, 95, 057205 10.1103/PhysRevLett.95.057205 16090916

[15] Lobo R. P. S. M., Moreira R. L., Lebeugle D., Colson D., Phys. Rev. B 2007, 76, 172105.

[16] Lee J. H., Fang L., Vlahos E., Ke X. L., Jung Y. W., Kourkoutis L. F., et. al, Nature, 2010, 466, 954 10.1038/nature09331 20725036

[17] Kimura T., Goto T., Shintani H., Ishiazaka K., Arima T., Tokura Y., Nature 2003, 426, 55 10.1038/nature02018 14603314

[18] Goto T., Kimura T., Lawes G., Ramirez A. P., Tokura Y., Phys. Rev. Lett. 2004, 92, 257201 10.1103/PhysRevLett.92.257201 15245056

[19] Kimura T.,Annu. Rev. Condens.Matter. Phys. 2012, 3, 93.

[20] Chun S. H., Chai Y. S., Oh Y. S., Jaiswal-Nagar D., Haam S.Y., Kim I., Lee B., et. al, Phy. Rev. Lett. 2010, 304, 0372041. 10.1103/PhysRevLett.104.037204 20366679

[21] Ishiwata S., Taguchi Y., Murakawa H., Onose Y., Tokura Y., Science 2008, 319, 1643 10.1126/science.1154507 18356519

[22] Rado G. T., Phys. Rev. Lett. 1964, 13, 335.

[23] Wang J., Neaton J. B., Zheng H., Nagarajan V., Ogale S. B., Liu B., et. al, Science 2003, 299, 1719 10.1126/science.1080615 12637741

[24] Momozawa N., Yamaguchi Y., J. Phys. Soc. Jpn. 1993, 62, 1292–1304.

[25] Tokunaga Y., Kaneko Y., Okuyama D., Ishiwata S., Arima T., Wakimoto S., et. al, Phys. Rev. Lett. 2010, 105, 257201 10.1103/PhysRevLett.105.257201 21231619

[26] Kimura T., Sekio Y., Nakamura H., Siegrist T., Ramirez A. P., Nature Mater. 2008, 7, 291. 18297078 10.1038/nmat2125

[27] Eerenstein W., Wiora M., Prieto J. L., Scott J. F., Mathur N. D., Nature Mater. 2007, 6, 348. 17417643 10.1038/nmat1886

[28] Tan G. L., Wang M., J. Electroceram. 2011, 26, 170.

[29] Tan G. L., Li W., J. Am. Ceram. Soc. 2015, 98, 1812.

[30] Sugimoto M., J. Am. Ceram. Soc. 1999, 82, 269.

[31] Yang N., Yang H., Jia J., Pang X., Journal of Alloys and Compounds 2007, 438, 263.

[32] Shen S. P., Wu J. C., Song J. D., Sun X. F., Yang Y. F., Chai Y. S., Nature Comm.

[33] Shen S. P., Chai Y. S., Cong J. Z., Sun P. J., Lu J., Yan L. Q., et. al, Phy. Rev. B 2014, 90, 180404.

[34] Rowley S. E., Chai Y. S., Shen S. P., Sun Y., Jones A. T., Watts B. E., et. al, Scientific Rep. 10.1038/srep25724 PMC4869023 27185343

[35] Tan G. L., Chen X. N., J. Elctron. Mater. 2013, 42, 906.

[36] Scott J. F., J. Phys.: Condens. Matter 2008, 20, 021001.

[37] Carver J.C., Schweitzer G. K., Carlson T. A., J. Chem. Phys. 1972, 57, 973.

[38] Grechnev G.E., Lyogenkaya A.A., Kotlyar O.V., Panfilov A. S., Gnezdilov V. P., Arxiv, 1510.00513V1, Cond-mat.mtrl-sci., 2. Oct. 2015.

[39] Mazumdar D., Knut R., Thöle F., Gorgoi M., Faleev S., Mryasov O. N., Shelke V., et. al, Journal of Electron Spectroscopy and Related Phenomena, 2016, 2068, 63.

[40] Pokharel B. P., Pandey D., J. Appl. Phys. 2000, 88, 5364.

[41] Stucki N., PhD thesis, P48, 2008, Université de Genève, Switzerland.

[42] Kenzelmann M., Harris A. B., Jonas S., Broholm C., Schefer J., Kim S. B., et. al, Phys. Rev. Lett., 2005, 95, 087206 10.1103/PhysRevLett.95.087206 16196899

[43] Hur N., Park S., Sharma P. A., Ahn J. S., Cheong S. W., Nature 2004, 429, 392 10.1038/nature02572 15164057

[44] Kimura T., Lawes G., Ramirez A. P., Phys. Rev. Lett., 2005, 94, 137201 10.1103/PhysRevLett.94.137201 15904022

[45] Wang J. L., Wang T. W., Li Y. Q., Guo H. X., Wang Q., 'Measurement for relative complex permeability by RF network /spectrum/ impedance analysis instrument', 259–263, Proceedings of 17th National Meeting on Electromagnetic Compatibility, Guangzhou, China, July 4 2007.

[46] Singh A. K., Snure M., Tiwari A., Patnaik S. J., Appl. Phys. 2009, 106, 014109.

[47] Ershov M., Liu H. C., Li L., Buchanan M., IEEE Trans. Electron. Dev. 1998, 45, 2196–2206.

[48] Bratkovsky A. M., Levanyuk A. P., Phys. Rev. B 2001, 63, 132103.

[49] Catalan G., Jiménez D., Gruverman A., Nature Mater. 2015, 14, 137–139. 25613711 10.1038/nmat4195

[50] Khan A. I., Chatterjee K., Wang B., Drapcho S., You L., Serrao C., et. al, Nature Mater. 2015, 14, 182–186. 25502099 10.1038/nmat4148

[51] Tan G. L., Sheng H. H., Acta Mater. 2016, 121, 144–151.

