# Supplementary material for: Magnetoelectric Response in Multiferroic SrFe12O19 Ceramics
Source: PLoS One. 2016 Dec 9;11(12):e0167084. doi: 10.1371/journal.pone.0167084 (PMC5147852; doi:10.1371/journal.pone.0167084)
Supplement: S1 File — (DOCX) [file pone.0167084.s001.docx]

**S1 File: SFO19 Supplementary File**

**1, Molecule Magnetic Moments**

The molecule magnetic moments were calculated for the measured B-H datasets for the SrFe_12_O_19_ ceramic with and without heat treatment in O_2_. The calculation process is given in the main manuscript. Here we present the calculation results of the molecule magnetic moments in *Figure A*.


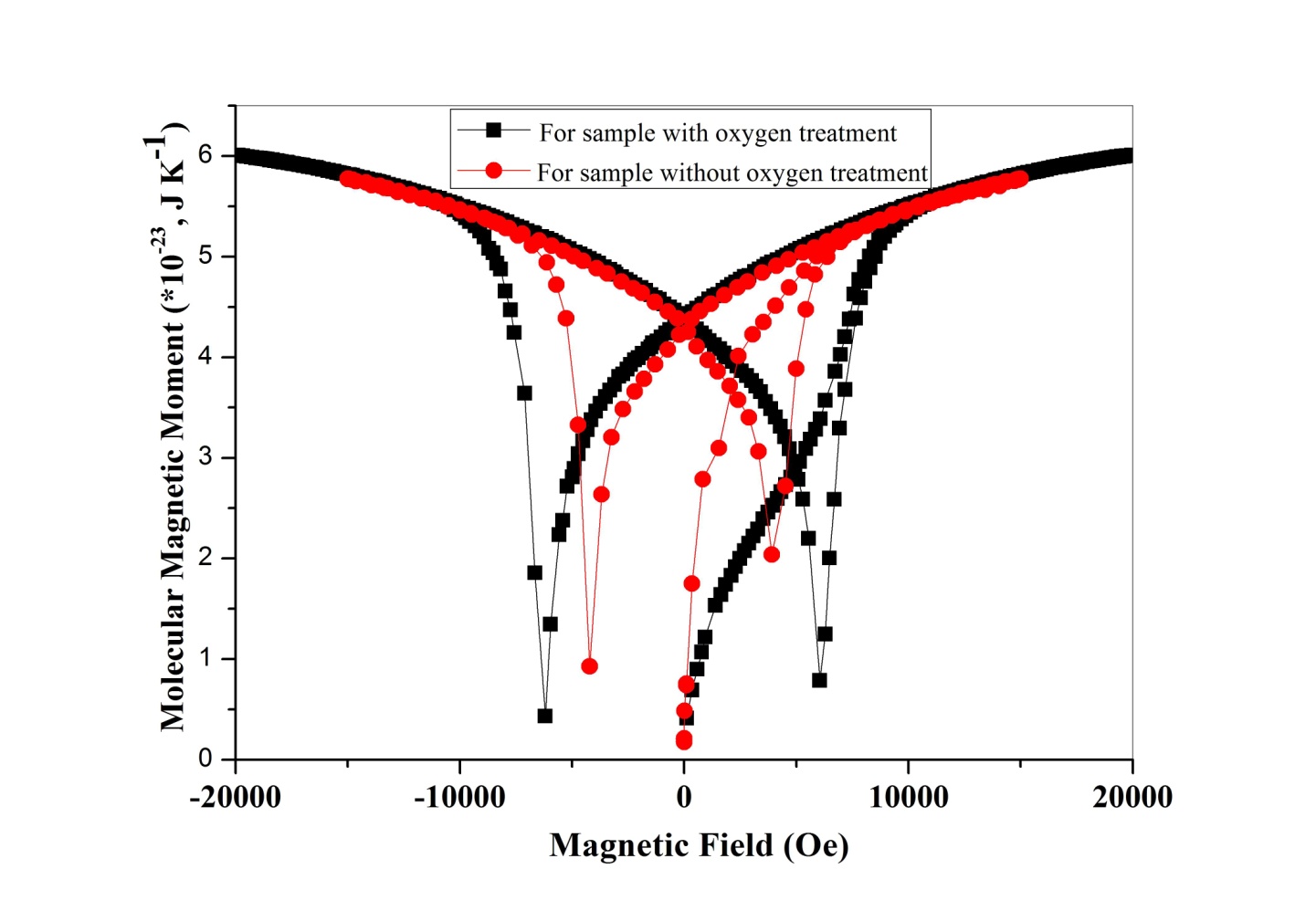


*Figure A: The plot of molecule magnetic moments as a function of magnetic field for SrFe_12_O_19_ ceramics (a) with and (b) without O_2_ heat treatment.*

**2，Magnetoelectric Coupling Effects of SrFe_12_O_19_ ceramics**

In order to check out if SrFe_12_O_19_ could generate coupling charge upon an external magnetic field. We set up a simple system for the ME coupling measurement, which was performed by measuring the output coupling voltage (V) as a function of the magnetic field (B). The SrFe_12_O_19_ ceramic was coated with silver electrodes on both sides and then placed in a space between two electromagnets. Upon the application of the magnetic field, the micro-voltmeter, which was linked with the electrodes on both surfaces of the sample, would output the variable coupling voltage with the external magnetic field B. *Figure B* shows such ME coupling voltages as a function of B at room temperature. By applying a low magnetic field from 0 to 50 mT, the coupling voltage varies from 0.04 mV to 0.1 mV, the coupling voltage changes very little within this range. With further increase of B from 50 mT to 200 mT, the coupling voltage shows a rapid enhancement from 0.1 mV to 3 mV, which then shows a slight decrease down to 2.2 mV when the magnetic field extends from 200 mT to 337 mT (*Figure B*). The variance ratio of the coupling voltage upon magnetic field B is around 7400%, which reflects a strong ME coupling effect. Then the coupling voltage exhibits a little fluctuation with a very small amplitude within the B range of 337 mT to 620 mT. Afterwards, with further increase of B up to 850 mT, the coupling voltage drops rapidly from 2.6 mV down to 0.3 mV (*Figure B*).


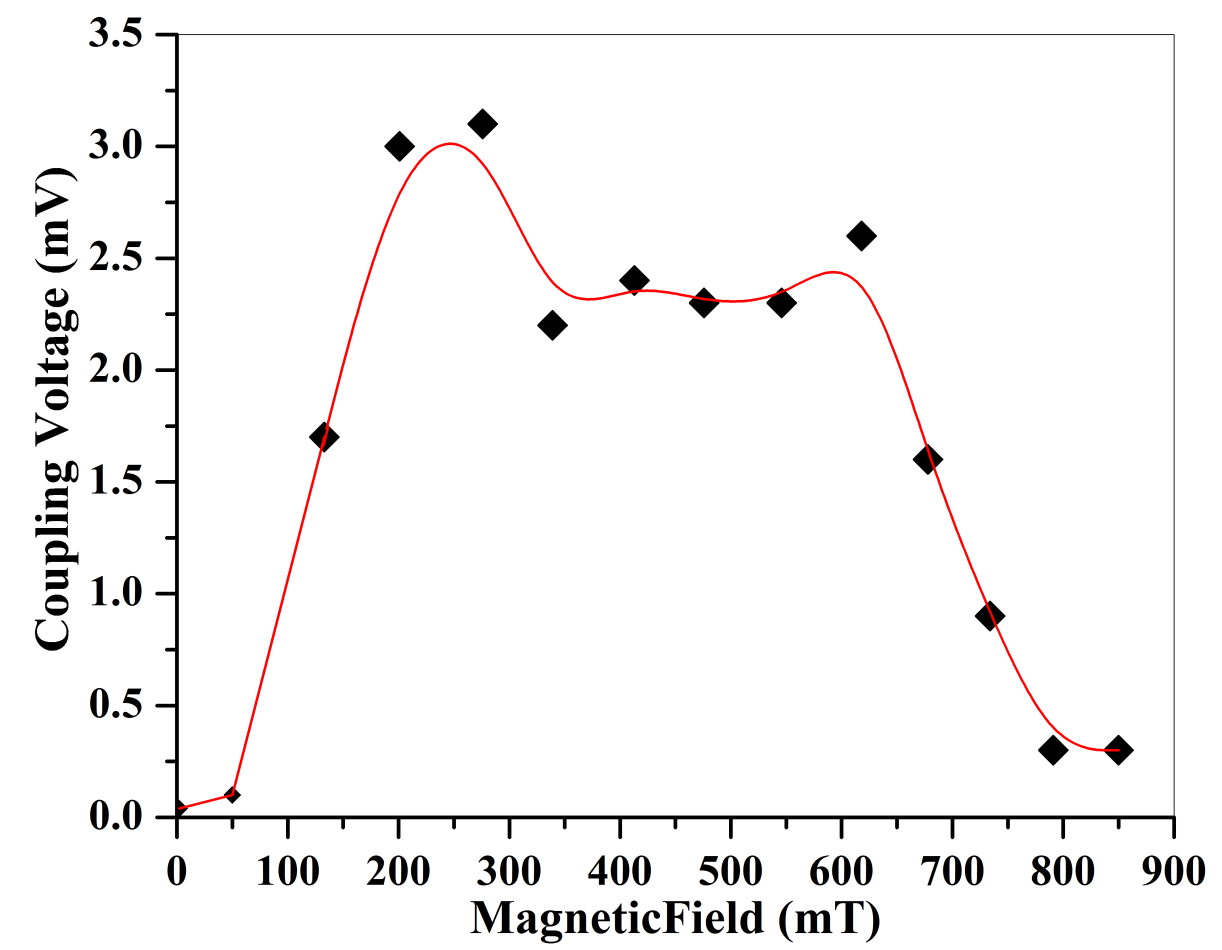


*Figure B: Plot of magnetoelectric coupling voltage as a function of applied magnetic field for SrFe_12_O_19_ ceramics.*

**3，Polarization Hysteresis Loops from Different Specimens**

**3.1 P-E Loop for non-O_2_ treated SrFe_12_O_19_ ceramic**


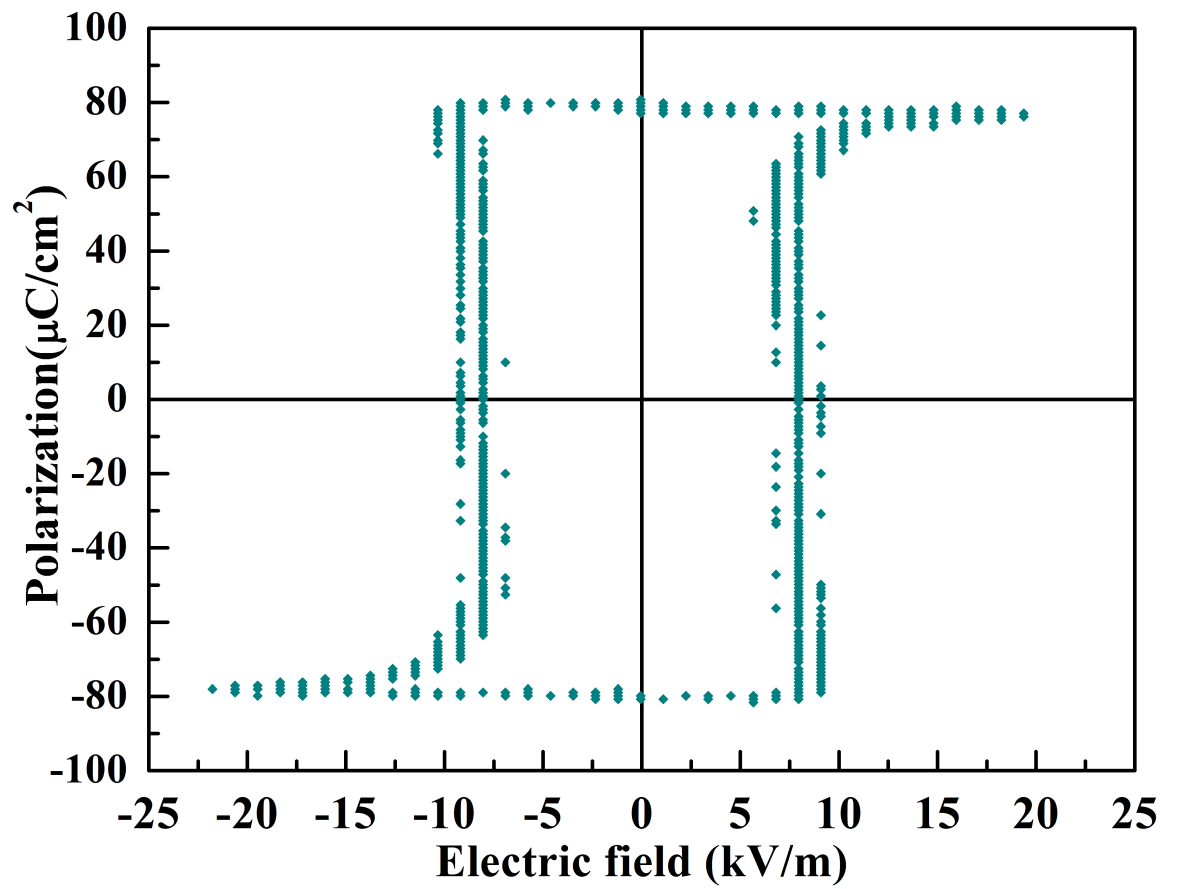


*Figure C: Ferroelectric polarization hysteresis (P-E) loop being measured on the second SrFe_12_O_19_ ceramic specimen, which was also sintered at 1150°C and subsequent heat-treatment in O_2_ for 9 hs with 3 steps wise.*

**3.2 P-E Loop for O_2_ treated SrFe_12_O_19_ ceramic**


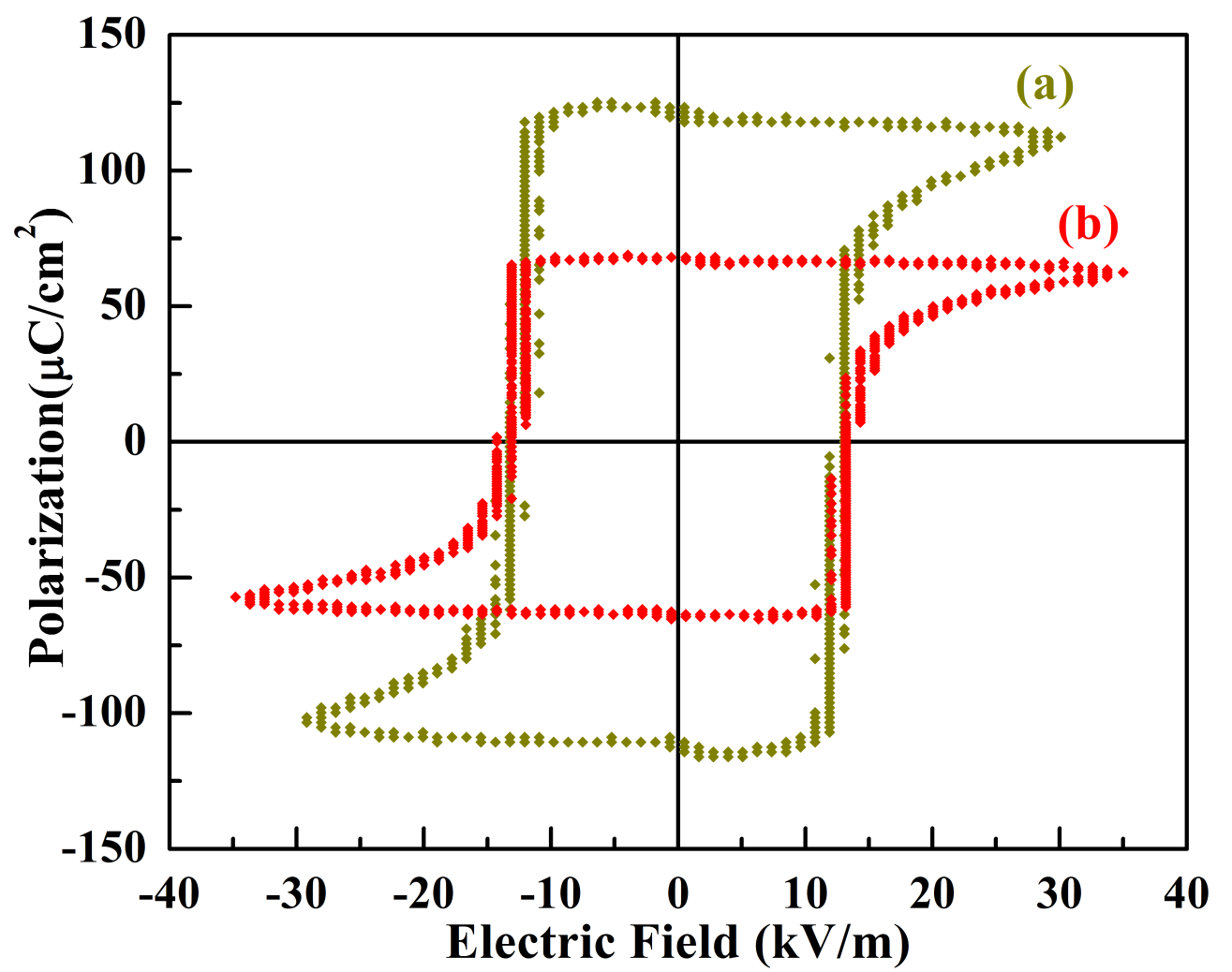


*Figure D: Ferroelectric polarization hysteresis (P-E) loop being measured on the third SrFe_12_O_19_ ceramic specimen with the same annealing process in oxygen for different applied field.*

**3.3 P-E Loop for non-O_2_ treated SrFe_12_O_19_ ceramic**





*Figure E: Ferroelectric polarization hysteresis (P-E) loop for SrFe_12_O_19_ ceramic, which was sintered at 1150°C for 1 hour only without subsequent heat-treatment in O_2_.*
